# Development of the first oligonucleotide microarray for global gene expression profiling in guinea pigs: defining the transcription signature of infectious diseases

**DOI:** 10.1186/1471-2164-13-520

**Published:** 2012-10-02

**Authors:** Ruchi Jain, Bappaditya Dey, Anil K Tyagi

**Affiliations:** 1 Department of Biochemistry, University of Delhi South Campus, Benito Juarez Road, New Delhi, 110021, India

**Keywords:** Microarray, Guinea pig, Transcriptional profile, Infectious diseases, Tuberculosis

## Abstract

**Background:**

The Guinea pig (*Cavia porcellus*) is one of the most extensively used animal models to study infectious diseases. However, despite its tremendous contribution towards understanding the establishment, progression and control of a number of diseases in general and tuberculosis in particular, the lack of fully annotated guinea pig genome sequence as well as appropriate molecular reagents has severely hampered detailed genetic and immunological analysis in this animal model.

**Results:**

By employing the cross-species hybridization technique, we have developed an oligonucleotide microarray with 44,000 features assembled from different mammalian species, which to the best of our knowledge is the first attempt to employ microarray to study the global gene expression profile in guinea pigs. To validate and demonstrate the merit of this microarray, we have studied, as an example, the expression profile of guinea pig lungs during the advanced phase of *M. tuberculosis* infection. A significant upregulation of 1344 genes and a marked down regulation of 1856 genes in the lungs identified a disease signature of pulmonary tuberculosis infection.

**Conclusion:**

We report the development of first comprehensive microarray for studying the global gene expression profile in guinea pigs and validation of its usefulness with tuberculosis as a case study. An important gap in the area of infectious diseases has been addressed and a valuable molecular tool is provided to optimally harness the potential of guinea pig model to develop better vaccines and therapies against human diseases.

## Background

Pioneering research of Robert Koch in the guinea pigs (*Cavia porcellus*) laid the foundation of microbiology and popularized this animal model for the study of various infectious diseases [1]. Since then, guinea pig has proven to be an indispensable tool in studying a range of infectious diseases, such as tuberculosis (TB) (*Mycobacterium tuberculosis*), anthrax (*Bacillus anthracis*), diphtheria (*Corynebacterium diphtheriae*), legionellosis (*Legionella pneumophila*), sexually transmitted diseases such as chlamydia infection (*Chlamydia trachomatis*), syphilis (*Treponema pallidum*) etc. [1]. These studies showed that guinea pigs bear substantial similarity to humans with respect to thymic and bone marrow physiology, innate immune responses, complement system, lung physiology, corticosteroid response, requirement of exogenous source of vitamin C and delayed-type hypersensitivity reaction to infections [1,2]. Due to these features, guinea pigs emerged as one of the best models for testing bio-defense agents and vaccines and for toxicological studies [1]. However, despite the tremendous contribution of this animal to medical research, the paucity of immunological reagents and molecular tools and the lack of fully annotated guinea pig genome sequence has severely hampered a holistic analysis of host responses in this model. Unlike the mouse model, gene deletion technology (for example, gene knockout and knock-in) and trans-gene expression is also unavailable in case of guinea pigs. Thus, considering the biological relevance of guinea pig model in a large number of infectious diseases, the importance of developing a comprehensive guinea pig microarray cannot be over emphasized. Recently, Tree and colleagues developed an oligonucleotide microarray comprising of 86 genes to study the host response to BCG vaccination in guinea pigs, however, despite its usefulness in immune response studies, it has limited applications due to a small number of genes in the array [3,4]. In this study, we have overcome this limitation by extending the cross-species hybridization technique to a number of mammalian species such as Human, Mouse, Rat, Macaque, Horse, Cat, Sheep, Pig, Chinchilla, Chimpanzee, Gray tailed opussum and Cattle in order to expand the number of features and have developed a 44 K guinea pig oligonucleotide microarray (GPOM). Further, to validate the array, the transcriptome of guinea pig lungs was analysed post - *M. tuberculosis* infection. Global gene expression profiling in this study not only facilitated the analysis of immunologically relevant genes but also helped in describing the transcriptional signature of pulmonary granulomas in guinea pigs represented by several key genes and commensurate pathways that are modulated in response to *M. tuberculosis* infection.

## Results

### Microarray design and annotation

**Figure 1:**
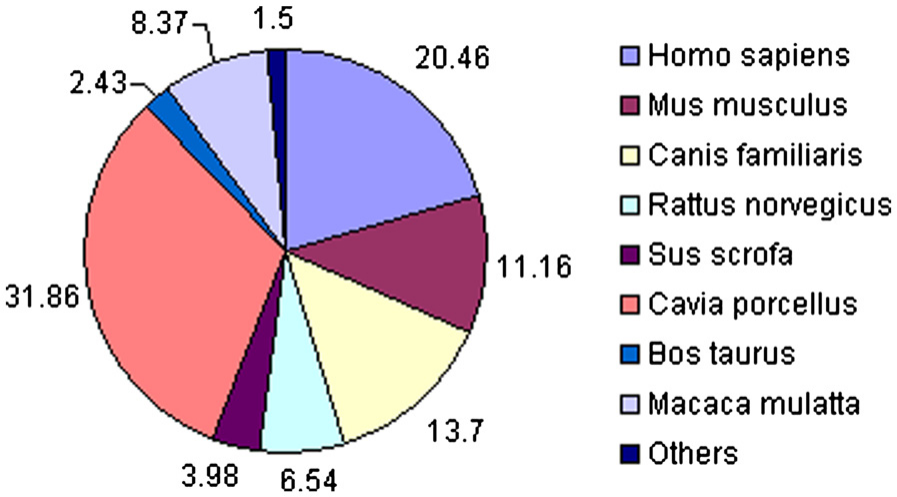
** Distribution of probes in 44 K GPOM.** The figure depicts the % distribution of oligonucleotide probes present in the 44 K guinea pig oligonucleotide microarray. The 60mer oligonucleotide probes were designed based on several mammalian species including human (*Homo sapiens*), mouse (*Mus musculus*), rat (*Rattus norvegicus*), rhesus monkey (*Macaca mulatta*), dog (*Canis familiaris*), horse (*Equus caballus*), cat (*Felis catus)*, sheep (*Ovis aries*), pig (*Sus scrofa*), chimpanzee (*Pan troglodyte)*, chinchilla (*Chinchilla lanigera*), gray-tailed opossum (*Monodelphis domestica*), cattle (*Bos taurus*) and guinea pig (*Cavia porcellus*). The % representation of a particular species is calculated with respect to the total number of probes in the array. The figure does not show some of the mammalian species separately for which, the % representation is < 1% and are collectively labelled as – others. Number wise distribution of probes from all the species is given in Table 1.

For gene expression profiling of species that lack genome sequence and/or representative microarray platforms, cross-species hybridization based microarray has conventionally been used. Since, fully annotated guinea pig genome sequence is not available, we employed cross-species hybridization technology to develop a 44 K microarray platform to study gene expression profile in guinea pigs. As described in the Additional file 1 and Supporting Table S1, initially a 244 K microarray was designed to contain 60 mer oligonucleotide probes from multiple mammalian species (human, mouse, rat, guinea pig, rhesus monkey, dog, horse, cat, sheep, pig, chimpanzee, chinchilla, gray-tailed opossum and cattle) based on all the probe sequences available from Agilent Catalogue arrays and NCBI mRNA sequences. Especially, the array included 1132 probes based on annotated gene sequences of guinea pig and 92,815 probes corresponding to guinea pig ESTs. The 244 K array was then hybridized with Cy3 labeled cRNA produced from pooled RNA obtained from various guinea pig tissues (lung, liver, spleen, brain, muscle, kidney and bone marrow) and Cy5 labeled genomic DNA isolated from guinea pig spleen tissue. Following hybridization, the array was scanned and features were extracted. The filtration criteria during the probe selection, while developing microarray by cross-species hybridization technology on Agilent platform, are based on comparison of specific signal intensity viz. the background signal intensity. Probes exhibiting significantly higher signal intensity (p < 0.05), at least 2 fold higher as compared to the background are selected for array development. Based on this criterion, a total of 20,023 out of 62,560 probes representing different mammalian genes were selected from the 244 K array. Similarly, a total of 9,823 out of 92,815 probes were selected for ESTs. However, irrespective of the intensities, all the 1,132 probes for guinea pig were included. Further, an additional of 12,825 best probes out of 19,975 newly added guinea pig EST’s from NCBI database were added to the 44 K array (Table 1). Thus, the final design of the guinea pig 44 K microarray comprised of a total number of 45,220 features including 29,846 valid features from different mammalian species (Figure 1), 1,132 probes for guinea pig transcripts and 12,825 probes for guinea pig ESTs, 1,264 Agilent positive controls and 153 Agilent negative controls. Agilent positive and negative controls are standard set of probes employed by the Agilent microarray platform for mammalian microarray studies. The negative control probes are intended to have no hybridization and these are used by feature extraction software for background determination. The positive controls are used to have predictable signals, which are used for monitoring the microarray linearity, sensitivity and accuracy. Based on the above-mentioned method for probe selection, many genes are represented by multiple but unique probes derived from different mammalian species. Use of multiple unique probes per transcript in general increases the confidence of microarray result. The averaging of the signals from multiple probes provides improved statistical confidence, reducing the impact of inconsistent probe behavior and improving the signal to noise ratio compared to the platforms that offer fewer probes per gene. For biological interpretation, homolog Gene ontology annotation was also obtained for all the probes by blast-based homology to reference sequence database of human, mouse and rat for which validated methods for biological pathway analysis are available.

**Table 1:** Probe distribution in 44 K GPOM: The table depicts the number of features derived from various mammalian species that have been used for designing the 44 K GPOM

**Organism**	**Sequence data source**	**Number of Probes in 44 K Array**
Human (*Homo sapiens*)	Agilent Catalogue Arrays	8964
Mouse (*Mus musculus*)	Agilent Catalogue Arrays	4889
Rat (*Rattus norvegicus)*	Agilent Catalogue Arrays	2863
*Rhesus Monkey (Macaca mulatta)*	Agilent Catalogue Arrays	3667
*Dog (Canis familiaris)*	Agilent Catalogue Arrays	6000
Horse (*Equus caballus*)	NCBI, mRNA sequences	186
Cat (*Felis catus*)	NCBI, mRNA sequences	64
Sheep (*Ovis aries*)	NCBI, mRNA sequences	164
Pig (*Sus scrofa*)	NCBI, mRNA sequences	1744
Guinea pig (*Cavea porcellus*)	NCBI, mRNA sequences	13957**
*Chinchilla lanigera*	NCBI, mRNA sequences	16
Chimpanzee (*Pan troglodyte*)	NCBI, mRNA sequences	201
Gray tailed opussum (*Monodelphis domestica*)	NCBI, mRNA sequences	25
Cattle (*Bos taurus*)	NCBI, mRNA sequences	1063
	**Total Number of probes**	**43803**

** 1132 + 12825 EST.

### Bacillary load and granulomatous pathology in the lungs of *M. tuberculosis* infected guinea pigs

**Figure 2:**
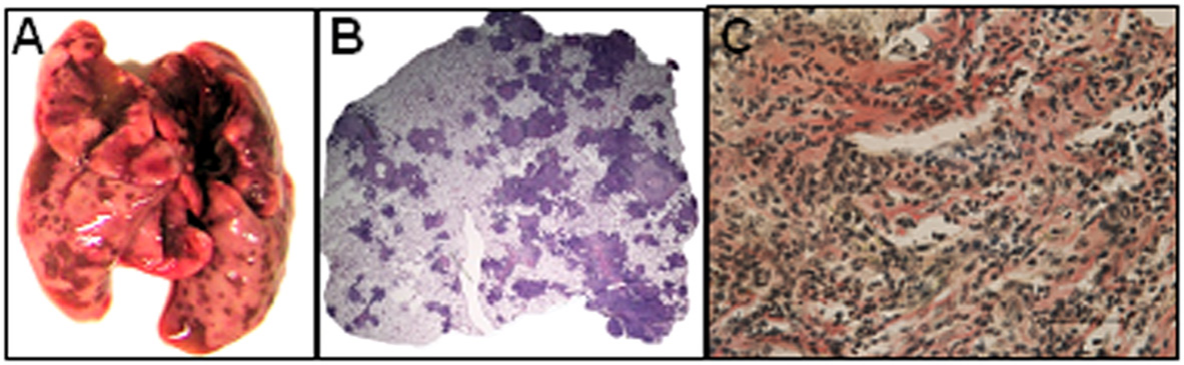
** Pulmonary pathology in *M. tuberculosis* infected guinea pigs.** The figure depicts (**A**) representative photograph showing gross pathology of lungs of *M. tuberculosis* infected guinea pigs at 10 weeks post-infection; (**B**) lower magnification photomicrograph of H&E stained, formalin fixed and paraffin embedded 5 μm section of lung tissue exhibiting multiple coalescing granulomas with central necrotic core and (**C**) photomicrograph (at 40X) of Van Gieson stained 5 μm lung section exhibiting extensive fibrosis as evident from thick bands of collagen (red color) around the granulomatous regions. Scale bar represents 1000 μm.

For gene expression analysis, RNA was isolated from lung tissues obtained from guinea pigs at 10 weeks following infection with ~500 tubercle bacilli. CFU analysis of guinea pig lungs at this time point revealed the presence of ~6.0 log_10_ tubercle bacilli/g of tissue. Moreover, infected guinea pigs exhibited severe pathological damage in lungs characterized by numerous large caseating granulomas encompassing central necrosis with occasional coalescence of multiple tubercles (Figure 2 A, B). In addition, extensive areas of irregular thick bands of fibrous collagen deposition were observed around the granulomas (Figure 2C). Hence, the gene expression in this phase of infection represents a transcription signature of advanced progressive TB disease.

### Gene expression profiling of pulmonary tuberculosis in guinea pigs by employing GPOM

We next compared the pulmonary gene expression profile of infected and uninfected guinea pigs by employing the GPOM developed in this study. As depicted in Additional file 2, clustered heat maps were obtained for all the genes on the 44 K GPOM in case of infected guinea pigs compared to uninfected control. Since, a small perturbation in the gene expression may considerably influence the biological response, a small difference in the fold change in gene expression are also relevant. Hence, genes exhibiting ≥1.5 fold difference in gene expression with a statistical significance of *p* < 0.05 were considered as differentially regulated. The rationale behind the selection of the cutoff for considerable fold difference while comparing the gene expression is based on standard practice in microarray data analysis [5-10].

**Figure 3:**
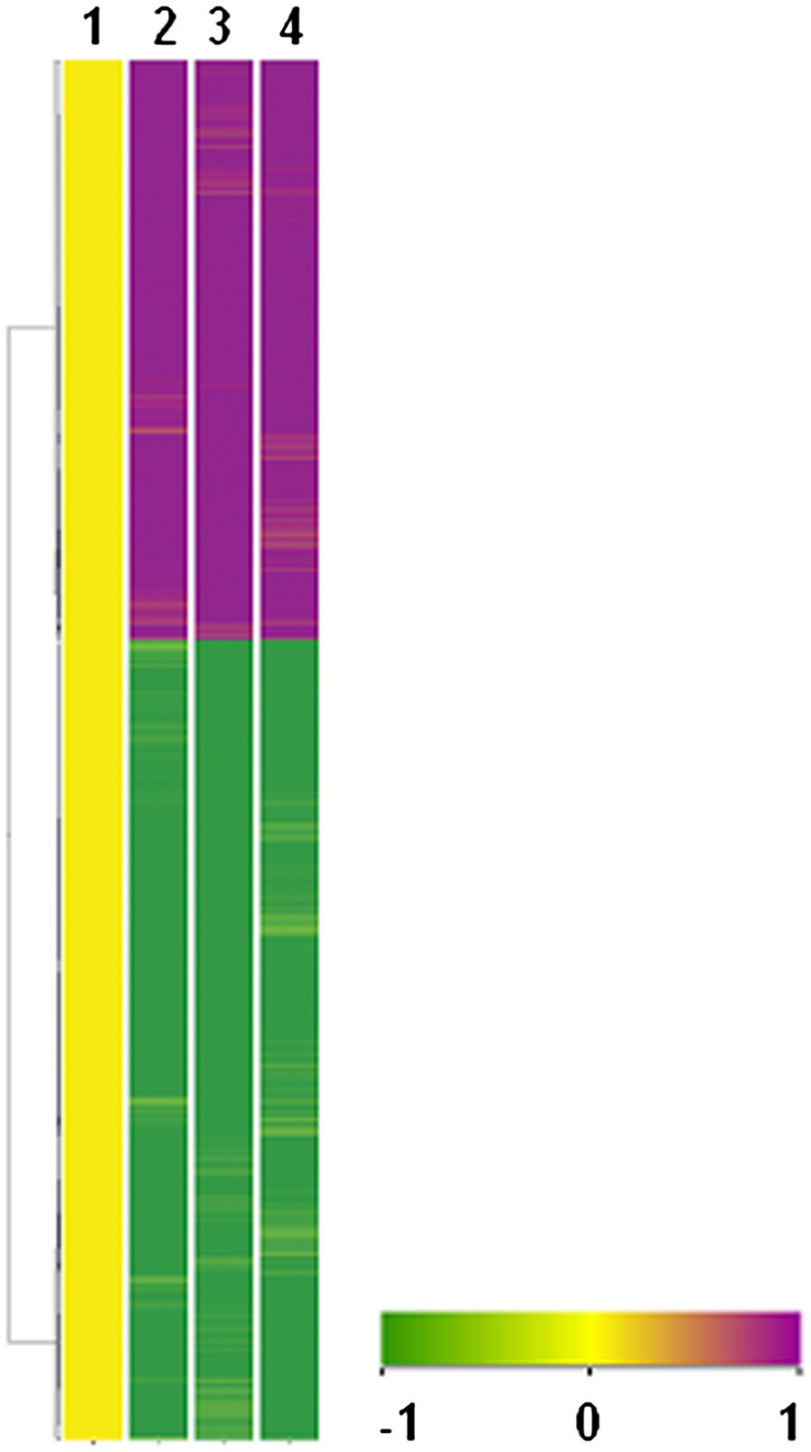
** Pulmonary gene expression signature of guinea pigs at 10 weeks post *M. tuberculosis* infection.** Transcriptional profile of lungs of guinea pig was analysed by microarray. The figure depicts the clustered heat maps obtained thereof for the genes expressed in a differential manner between the experimental and control groups. By using unsupervised hierarchical clustering algorithm, the most similar expression profiles are joined together to form a group. These are further joined in a tree structure, until all data forms a single group. Clustering is based on Average- distance between two clusters, which is the average of the pair-wise distance between entities in the two clusters. For the measurement of similarity between conditions, Pearson coefficient correlation clustering algorithm is used. The color scheme for the hierarchical clustering is yellow: no change in expression, magenta: higher expression in infected lungs relative to normal lungs and green: lower expression in infected samples relative to normal uninfected lungs. 1: Uninfected lung; 2: Infected Lung 1; 3: Infected Lung 2; 4: Infected Lung 3.

Based on this, several unique genes were identified that exhibited a significant regulation in response to infection. While, 1344 unique genes exhibited a marked up regulation, 1856 genes were significantly down regulated in the lungs of infected guinea pigs as compared to the lungs of uninfected animals as depicted by a heat map in Figure 3. The genes exhibiting significant regulation are listed in Additional file 3.

Differentially regulated genes were further classified in to different categories based on their direct or indirect involvement in various biological processes or pathways. Based on this categorization, a significant alteration was observed in the expression of several important genes related to metabolic pathways, cell signaling, immune response and other miscellaneous functions (Additional file 4). Some of the key pathways are listed in Tables 2, 3 and 4.

**Table 2:** **Regulation of key metabolic pathways in guinea pig lungs infected with *M. tuberculosis***

**Pathway**	**Up**	**Down**
**Oxidative Phosphorylation**	NDUFA12, NDUFV1, UQCRC2, SDHB, NDUFAB1, ATP5C1, PPA1, ATP6V0E1, ATP5J, NDUFB8, ATP5A1, NDUFA7, COX5A, LHPP, UQCRC1, ATP5G3, NDUFA11, ATP5J2, NDUFV3, NDUFV2, NDUFS6, NDUFS8, NDUFS3, ATP6V1D, COX5B, COX6A2, COX4I1, ATP6V1F, ATP6V0D1, COX7A2L, ATP6V0C, ATP6V1E1, ATP6V0B, ATP6V1B2, NDUFB4, NDUFB2, NDUFA3, COX17	ATP5O, ATP5F1, ATP12A, NDUFB5, NDUFB7, NDUFA4
**Glycolysis/Gluconeogenesis**	LDHA, ENO1, HK1, ADH5, TPI1, GAPDH, ADH1A, AKR1A1, ALDOA, PGK2, ENO3, GPI	ALDOC, DLAT, PFKL
**TCA cycle**	FH, SDHB, PC, MDH2, IDH1, ACO2, SUCLG1	DLAT
**Lipid, Glycerolipid and Glycerophospholipid metabolism**	ABHD2, ABHD8, ACSL5, ADH1A, ADH5, AGPAT2, AKR1A1, CPT2, CEPT1, CYP27A1, CYP2B18A, CYP26B1, DGKA, FDPS, GCDH, HADHA, HADHB, LPL, PLA2G2A, PGS1	AGPAT1, AGPAT3, ACSL1, CPT1B, CYP2F1, CYP2A13, CYP1A2, CYP11A1, CYP2D40, PLD2, PPAP2A, PLA2G2C, PLA2G5, PAFAH1B1, PNPLA3, PTDSS2, LPGAT1
**Purine and Pyrimidine metabolism**	ADSS, NT5C2, NPR2, AK2, XDH, ADCY4, POLR2E, HPRT1, PRP, DPYD, TXNRD2, NT5C2, CAD	AK1, DCTD, POLA1, POLR2F, POLR2K, PRPS1L1, GUK1, PDE6G, PDE6A, POLR3GL, ENPP1, ZNRD1, PRUNE, TYMS, TK1
**Amino acid metabolism**	GLUL, GCDH, HADHA, HADHB, PLOD3, TMLHE, SETD1A, SETD8, HSD17B10, IVD, LDHA, MIF, PRDX6	ALDH4A1, ALDH6A1, ASS1, AMT, AGXT, AGMAT, ABAT, AHCYL1, CYP1A2, CKB, DNMT1, DBT, DDC, GLS, GLUD1, GLDC, GNMT, HMGCS1, NOS1, MAT2A, ODC1, WHSC1L1, SUV420H2, SUV420H1

**Table 3:** **Regulation of key signaling pathways in guinea pig lungs infected with *M. tuberculosis***

**Pathway**	**Up**	**Down**
**MAPK**	MRAS, MAPK7, RAC1, TGFB1, MAPKAPK5, MAPKAPK2, FGFR3, IL1B, PLA2G2A, RAC2, RAP1B, SOS2, MAPK8IP3, RRAS, HSPB1, CACNA1E, ARRB2, MAP2K2, FGF17, STK4, SRF, PAK2	MEF2C, CACNB2, CACNB4, TAOK3, CACNG4, DUSP9, FGFR4, PPP5C, FGFR2, FGFR1, RAP1A, CACNA1C, FOS, TNF, RPS6KA1, TGFB3, PPM1A, FGF19, ATF4, AKT3, CRKL, MAPK11, FGF8, MAPK1, FGF2, TP53, PPP3CB, MAPK8IP2, FGF12, FGF10, MAP3K4, PLA2G2C, PLA2G5, PRKACG, PRKACA, CHUK, PTPRR, RASA1, CACNA1S, BRAF, MAP2K6, MAP2K1, DUSP2, DUSP4, RASGRP2
**Wnt**	RAC1, CUL1, CSNK2A2, PPP2R1A, RAC2, CSNK1A1, PPP2CA, RHOA, PSEN1	PRICKLE1, CSNK1E, LRP5, PLCB3, EP300, TBL1XR1, CTBP1, WNT3, CAMK2G, CAMK2A, CCND1, NKD2, SMAD4, SMAD2, PPP2R5D, TP53, PPP3CB, CSNK2A1, CTNNB1, PRKACG, PRKACA, CAMK2D, LEF1, WNT10A, FBXW11, BTRC, FZD9, FZD3, TCF7L2
**Calcium**	VDAC1, ERBB2, ATP2A1, GNAQ, TNNC1, CACNA1E, P2RX2, ADCY4, SLC25A6	PLCB3, ADRA1B, PHKG2, CACNA1C, ADORA2A, CAMK2G, CAMK2A, GNAS, PLN, HTR6, NOS1, RYR1, VDAC2, PPP3CB, SLC8A1, CYSLTR1, P2RX1, CALM2, ATP2B4, ATP2A3, PRKACG, PRKACA, CAMK2D, LTB4R2, CACNA1S, ADRB2, PLCG1

**Table 4:** **Regulation of key immune response related genes in guinea pig lungs infected with *M. tuberculosis***

**Pathway**	**Up**	**Down**
**Chemokine signaling pathway**	GNB4, RAC1, IL8, GNG5, HCK, RAC2, FOXO3, RAP1B, SOS2, ADRBK1, NCF1, GNAI2, CCL27, VAV3, RHOA, ADCY4, ARRB2, NFKBIB	GNB1, PLCB3, CCL5, CXCL9, RAP1A, CXCR3, GNG8, GNG2, CCL21, AKT3, CRKL, MAPK1, PRKACG, PRKACA, CHUK, CCL11, BRAF, MAP2K1, RASGRP2
**Cell adhesion molecules (CAMs)**	HLA-DQB1, ITGB7, HLA-A, HLA-B, HLA-B, HLA-DMA, NRXN2, HLA-DPA1, HLA-DRB1, HLA-DRB1, ALCAM, CLDN18, CLDN14	MPZL1, HLA-E, HLA-F, CADM3, CADM1, CD34, CD8A, CD99, CDH3, CLDN4, CLDN6, NCAM1, GLG1, NLGN2, ITGB2, ITGB8, ITGA9, CNTNAP1, SIGLEC1
**Cytokine-cytokine receptor interaction**	IL1, RAP, IL8, TGFB1, IL1B, CSF1R, IL23A, BMP8A, CCL27, BMP4, BMPR2	GHR, CCL5, CD70, CXCL9, KIT, CD27, CXCR3, TNFα, IL2RG, CNTFR, BMPR1A, IL23R, CCL21, TGFB3, FLT4, BMP5, IFNGR2, LTA, IL9, CCL11, TNFSF10, ACVR2A
**Leukocyte trans-endothelial migration**	RAC1, CYBA, CYBB, RAC2, RAP1B, ACTG1, NCF1, GNAI2, EZR, ACTN4, VAV3, RHOA, PTPN11, CLDN18, CLDN14, ACTB	RAP1A, CD99, RHOH, THY1, MAPK11, CTNNB1, CLDN4, CLDN6, ITGB2, MLLT4, PLCG1
**Natural killer cell mediated cytotoxicity**	RAC1, ARAF, HLA-A, HLA-B, RAC2, SOS2, VAV3, LAT, FCER1G, PTPN11, MAP2K2, PTPN6	TYROBP, HLA-E, HLA-F, TNF, IFNGR2, MAPK1, PPP3CB, GZMB, ITGB2, TNFSF10, BRAF, MAP2K1, PLCG1
**T cell receptor signaling pathway**	SOS2, GRAP2, VAV3, LAT, RHOA, NFKBIB, MAP2K2, PTPN6, PAK2	CD8A, FOS, TNF, NCK1, AKT3, MAPK11, MAPK1, PPP3CB, CHUK, MAP2K1, PLCG1
**B cell receptor signaling pathway**	RAC1, RAC2, SOS2, CD81, VAV3, LILRB3, NFKBIB, MAP2K2, PTPN6, CR2	FOS, AKT3, MAPK1, PPP3CB, CHUK, MAP2K1
**Antigen processing and presentation**	HLA-DQB1, HLA-A, HLA-B, HLA-B, CD74, HLA-DMA, HLA-DPA1, HLA-DRB1, HLA-DRB1, HSP90AB1, CTSB, CTSS, HSP90AA1	HLA-E, HLA-F, CD8A, TNF, TAPBP, HSPA5, PSME2
**Complement and coagulation cascades**	C4A, C3, C1S, SERPING1, CR2	CD55, MASP2, KNG1, SERPINC1, F8, C9, C6, C3AR1, C5AR1

Pathway analysis revealed that more than 196 genes that were differentially regulated in infected samples as compared to the uninfected control samples were associated with general metabolism (Additional file 4). In particular, several key genes (Table 2) involved in oxidative phosphorylation (44 genes), purine and pyrimidine metabolism (28 genes, respectively), amino acid metabolism (37 genes), glycolysis/gluconeogenesis (15 genes), glycerophospholipid and glycerolipid metabolism (13 and 8 genes, respectively), fatty acid (9 genes) and arachidonic acid metabolism (9 genes), pentose phosphate pathway (9 genes), TCA cycle (8 genes) and ether metabolism (7 genes) were significantly regulated in response to infection (Additional file 4).

Among the cell signaling pathways, the most profound modulation was observed in the MAPK signaling pathway with perturbation of 67 genes (Table 3). In addition, the expression of several key genes related to Wnt (38 genes), calcium (36 genes), neurotrophin (34 genes), insulin (28 genes), TGFβ (25 genes), ErbB (18 genes), JAK-STAT (17 genes), PPAR (15 genes), VEGF (15 genes), hedgehog (14 genes), p53 (11 genes), notch (8 genes) and phosphatidylinositol signaling systems (4 genes) were also affected (Additional file 4). Characteristically, majority of the genes related to MAPK, Wnt and Calcium signaling pathways exhibited a marked down regulation (Table 3).

Among the immune response related genes, a significant alteration was observed in the expression of several key genes associated with chemokine signaling pathway (37 genes), cell adhesion molecules (CAMS) (32 genes), cytokine-cytokine receptor interaction (33 genes), leukocyte trans-endothelial migration (27 genes), natural killer cell mediated cytotoxicity (25 genes), T cell receptor signaling (20 genes) and B cell receptor signaling (16 genes) (Table 4). In addition, profound modulation was observed in genes associated with the antigen processing and presentation (20 genes), hematopoietic cell lineage (16 genes) and complement and coagulation cascades (14 genes) (Table 4 and Additional file 4). A marked regulation was also observed in the signaling pathways related to TGF-β (25 genes), Toll-like receptor (19 genes), Fc epsilon RI (18 genes), adipocytokine (13 genes), NOD-like receptor (13 genes), RIG-I-like receptor (12 genes), mTOR (11 genes) and intestinal immune network for IgA production (8 genes) (Additional file 4).

### Validation of microarray results by real time RT-PCR

For the validation of 44 K GPOM, 5 genes were selected based on the following criteria: (i) differential regulation, (ii) immunological relevance and (iii) availability of cDNA sequence in the NCBI database. Expression of these genes was analyzed on the same RNA samples, which were used for the microarray study by semi- quantitative real time RT-PCR by employing SYBR green PCR Master Mix (Applied Biosystems) with 18S as the internal control. The primer sequences of C3AR1, CAMP, CCL5, IFNγ, C4BPA and 18S rRNA genes were designed using the guinea pig gene specific cDNA sequences available in the public database (NCBI) based on the recommended guidelines for designing real time PCR primers (Primer express software, Applied Biosystems]. Sequences of the primers are described in Supporting Table S2 in Additional file 1. The primers were experimentally validated for two standard quality control criteria, (i) single amplicon specificity by analysis of dissociation curves and (ii) consistently high amplification efficiency by analysis of calibration curve. For analysis of real time PCR data ΔΔCt method was employed [1]. Consistent with our microarray results, wherein several probes corresponding to C3AR1, CAMP, CCL5 and IFNγ exhibited a down regulation along with up regulation of C4BPA, real time PCR results also matched the microarray expression profile (Supporting Table S2 in Additional file 1).

## Discussion

Guinea pig model has made tremendous contribution towards the understanding of several infectious and non-infectious human diseases [1,2]. Moreover, in case of TB, guinea pig has emerged as the most preferred and biologically relevant model to investigate TB pathogenesis and therapy [11]. The success of *M. tuberculosis* as an intracellular pathogen is primarily attributable to its ability to reside in human lungs inside hypoxic granulomas in a dormant stage for years or even decades. However, the conventional mouse model for TB does not form hypoxic granulomas, which serve as the primary host defense mechanism for the containment of infection and is the central feature of TB pathogenesis in humans. Thus, due to its ability to produce hypoxic granulomas guinea pig as a surrogate model fulfills an important niche in the field of TB. However, the lack of a fully annotated genome sequence remains a major impediment towards the realization of full potential of guinea pig as an animal model. Moreover, till date not even a single study has investigated the genome-wide transcriptional response associated with *M. tuberculosis* infection in guinea pigs. Thus, development of a microarray platform to understand the host responses in guinea pigs is a critical step towards harnessing the true potential of this model.

In the present study, by employing cross-species hybridization technique, we have developed an oligonucleotide microarray with 44,000 features assembled from different mammalian species, which to the best of our knowledge is the first attempt to employ microarray to study global gene expression profile in guinea pigs. In order to demonstrate the utility of this microarray and to gain insight into the host responses, we have carried out the expression profile of guinea pig lungs at 10 weeks post-infection with *M. tuberculosis*. At this stage of infection, guinea pigs exhibit advanced stage of TB disease with multiple coalescing granulomas along with caseation and liquefaction necrosis in the lungs [2]. Hence, the gene expression profile observed in this study represents a transcriptional signature of advanced progressive TB disease in guinea pigs*.*

**Figure 4:**
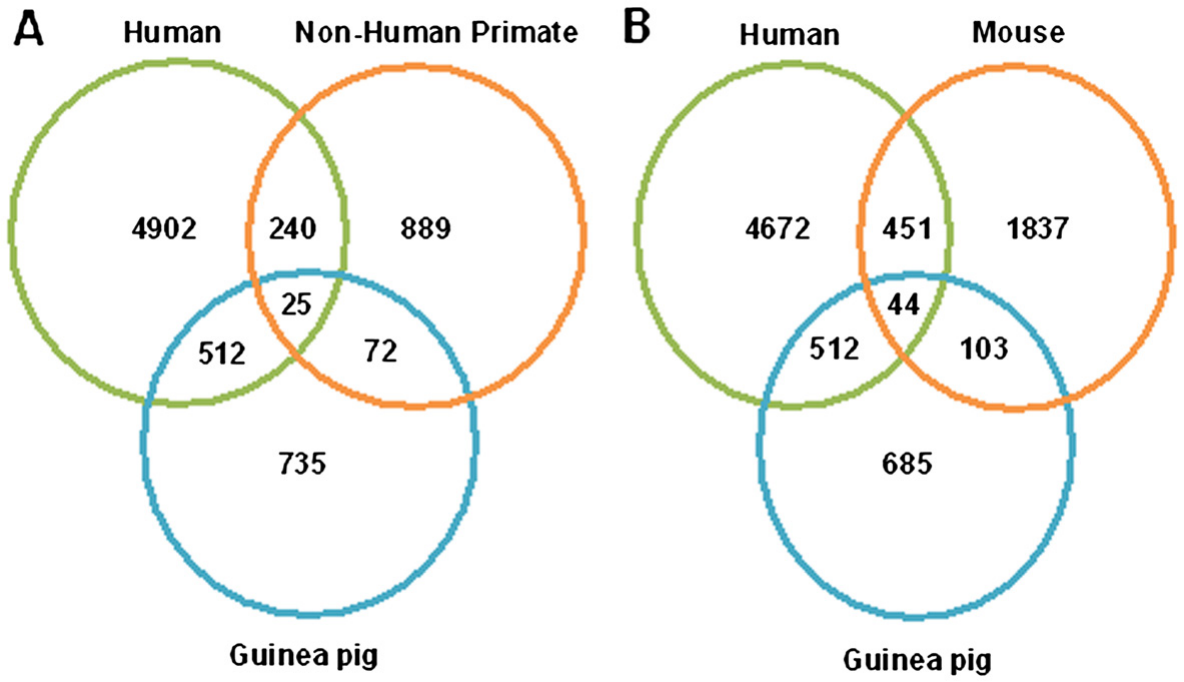
** Comparison of transcriptional response of guinea pig, human, non-human primate and mouse to *M. tuberculosis* infection.** The Venn diagrams depict the degree of overlap between up regulated genes of (**A**) Guinea pig, human and non-human primates and (**B**) Guinea pig, human and mouse. The analysis included comparison of the list of differentials obtained from our study with that obtained from various microarray data available in the public database for TB infection in case of; human [GEO Accession no. GSE20050], mouse [GEO Accession no. GSE15335] and non-human primate [GEO Accession no. GPL10183]. Down regulated genes did not show any considerable overlap across the species, hence not depicted in the figure.

In our study, the pulmonary transcriptional profiling of *M. tuberculosis* infected guinea pigs revealed a significant regulation of 3200 unique targets. While, 1344 unique genes exhibited a marked up regulation, 1856 genes were significantly down regulated. Differentially regulated genes were further classified into different categories based on their direct or indirect involvement in various biological processes or pathways. A massive re-alignment of metabolic pathways, mostly associated with catabolism, emerged as one of the interesting themes from this analysis. Although, altered metabolic functions of the host have earlier been reported in human subjects and in laboratory animals in response to febrile infections involving wasting of body tissues [12], most studies have been restricted to biochemical and *in silico* analysis and have not looked beyond immune mechanisms in order to probe the underlying cause of pathology and active disease in case of TB [13,14]. Only recently, Mi-Jeong and colleagues reported a correlation between caseation of human TB granulomas with elevated host lipid metabolism in these infected tissues by microarray [15]. Extensive necrosis observed in the pulmonary granulomas in our study as well as a marked up regulation of several of these lipid homeostasis related genes, such as, ABHD2, ABHD8, ACSL1, ACSL5, CYP27A1, CYP2B18A, CYP26B1, CYP2F1, CYP2A13, CYP1A2, CYP11A1, CYP2D40, CYP2F1, FDPS, HADHA and LPL correspond well with the observations associated with human caseous granulomas [15]. On comparing the entire list of up and down regulated genes from our guinea pig study with that obtained from human TB granuloma study [GEO Accession no. GSE20050] [15], we observed that 38% of the up regulated genes of guinea pig [512 out of 1344 genes] exhibited an overlap with the genes up regulated in humans (Figure 4). Further, on comparing the microarray data available in the public database for TB infection in case of humans [GEO Accession no. GSE20050] [15], mouse [GEO Accession no. GSE15335] [16] and non-human primates [GEO Accession no. GPL10183] [17], while, the non-human primates and humans exhibited a 19% overlap between up regulated genes, the overlap between mouse and humans was 18% (Figure 4 and Additional file 5). The guinea pig model is known for its close similarity to humans in terms of pathological response to *M. tuberculosis* infection [2]. Our observations indicate that guinea pigs also exhibit higher resemblance to humans in terms of transcriptional response to *M. tuberculosis* infection, which further validates it as an excellent animal model to study TB. Hence, findings of this study would have a direct implication towards the development of novel therapeutic interventions. Besides, it would also permit the development and validation of biomarkers for effective vaccines and drugs in guinea pig model. 

Induction of catabolic processes with consequential ATP accumulation has recently been shown to provide an interface between metabolism and host defense to infection [18-20]. ATP molecules generated in response to injury to airway epithelial cells have been reported to be a critical determinant of cell migration and repair following the injury and have been shown to be associated with the activation of down-stream signaling cascades and induction of IL-1β through the interaction of ATP with purinergic receptors [21]. A few *in vitro* studies have also indicated the role of ATP mediated macrophage apoptosis in killing of *M. tuberculosis*[19]. A concurrent up regulation in the expression of oxidative phosphorylation related genes (expected to result in increased ATP levels), purinergic receptors and IL-1β in this study to the best of our knowledge, provides the first *in vivo* evidence for the involvement of these pathways in TB. Further, the lungs of the infected guinea pigs also exhibited a marked perturbation in the expression of several key genes associated with chemokine signaling (CCL27, CCL5, CXCL9, CXCR3, CCL21 and CCL11), cell adhesion molecules (CAMs) (HLA, ALCAM, MPZL1, CADM3, CADM1, CD34, CD8A, CD99, CDH3, CLDN4, CLDN6, NCAM1, ITGB2, ITGB8 and ITGA9) and cytokine and cytokine receptors (IL1β, IL1RAP, IL2RG, IL8, IL9, IL23A, IL23R, TGFB1, TGFB3, IFNGR2, TNFα, TNFSF10, CSF1R, BMP4, BMP8A, BMPR1A, BMPR2, LTA and ACVR2A), which are known to contribute to leukocyte trans-endothelial migration, inflammation and granulomatous pathology.

Perturbation in the cellular signaling pathways is another typical theme that emerged from our study. The most prominent observation relates to the repression of numerous genes related to MAPK, Wnt and calcium signaling pathways. These observations are consistent with previous studies, which have suggested that modulation of MAPK signaling pathway along with the reduction in the levels of intracellular calcium are some of the important means by which, *M. tuberculosis* represses phagosome - lysosome maturation and pro-inflammatory responses at the site of infection [22,23]. MAPK signaling is known to be crucial for the anti-bacterial response of the host and it also represents a strategic target for bacterial subversion tactics [24]. Thus, dampening of the MAPK signaling has emerged as a key to achieve alteration in the antibacterial phenotype of macrophages. Recently, Wnt signaling pathway has been implicated in the generation of long-lived multi-potent memory T cells and in the modulation of inflammatory response of macrophages to *M. tuberculosis* infection [25,26], thus repression of Wnt signaling pathway observed in this study suggests a possible mechanism by which, *M. tuberculosis* inhibits effective T cell memory response.

Another key observation from this study relates to the modulation of several key genes involved in the re-modeling of extracellular matrix such as, COL6A2, COL14A1, COL12A1, MMP24, TIMP1, SERPING1, SERPINB1, ADAMTS1, ADAMTS7, MMP1, PITRM1, SERPINA3N, SERPINB6, SERPINE2, SERPINH1 and CNDP2. These observations are in agreement with the previous studies, which have implicated these genes in dual events associated with tissue remodeling as well as tissue damaging [27-30]. The presence of extensive necrosis along with thick bands of collagen observed in the guinea pig pulmonary granulomas indicates that the balance is heavily tipped in the favor of tissue damaging events. Increased expression of the complement receptor CR2 and numerous key genes involved in phagocytosis and antigen presentation as observed in this study further substantiates that the pathogen exploits the normally effective defense system to its advantage by subverting or co-opting these pathways as has been also reported earlier [17,22].

## Conclusion

This study reports for the first time the development of a 44 K oligonucleotide microarray for guinea pigs and provides an important tool to capture the genome wide transcriptional changes in this model. The transcriptional profiling of *M. tuberculosis* infected guinea pig lungs not only revealed modulation of key immunologically relevant genes but also demonstrated involvement of novel metabolic and signaling pathways in TB pathogenesis. Moreover, *in silico* analysis revealed a higher resemblance of guinea pigs to humans in terms of transcriptional response to *M. tuberculosis* infection when compared to mouse and non-human primates. Development of the 44 K GPOM is thus, a critical step towards characterization of the guinea pig model, which will greatly aid in improving our understanding of host responses to a number of infectious diseases. We believe that optimal use of guinea pig model and further research on its biology would generate tremendous opportunity to understand host-pathogen interaction and thus, help in the development of new therapeutic intervention strategies.

## Methods

### Ethics statement

Guinea pig experiments were reviewed and approved by the Institutional Animal Ethics Committee of University of Delhi South Campus, New Delhi, India (Permit number: 159/1999/CPCSEA). All procedures with the infected animals/tissues were performed in a Biosafety level three (BSL-3) containment facility at the University of Delhi South Campus, according to the approved protocols. All animals were routinely cared for according to the guidelines of CPCSEA (Committee for the Purpose of Control and Supervision on Experiments on Animals), India with a 12 hr light/dark cycle (0600–1800) maintained at 25 degrees Celsius with a relative humidity of 50%.

### Experimental animals, infection and study design

Pathogen free 6–8 weeks old (200-300 g) female outbred guinea pigs (Dunkin Hartley strain) used for the microarray studies were procured from Disease Free Small Animal House Facility, CCS Haryana Agricultural University, Hisar, India. The infected and uninfected animals were housed separately in individually ventilated cages (2 animals/cage) and were provided with *ad libitum* food and water in the BSLIII facility at University of Delhi South Campus, India. Guinea pigs were infected by using the method as described previously [31]. Briefly, *M. tuberculosis* (H37Rv strain, ATCC no. 25618 procured from AIIMS, New Delhi, India) was grown to mid-log phase in Middle Brook 7 H9 media (0.05% Tween_80_ and 0.5% Glycerol) and stocks were prepared as described [32]. The CFU of stocks was enumerated by plating 10 fold serial dilutions on 7 H11 agar (1XADC and 0.5% glycerol). By using pre-calibrated infection parameters, guinea pigs were infected in the inhalation exposure system, (Glas-col Inc.), which resulted in ~500 bacilli in the lungs of guinea pigs at day 1 post-infection. For enumeration of day 1 CFU, the whole lung homogenates were plated onto 7 H11 agar plates and colonies were counted after 3 weeks of incubation at 37°C.

### Necropsy procedure and histopathological evaluation

Guinea pigs were euthanized by carbon dioxide asphyxiation. After aseptically dissecting the animals, for histopathological evaluation, three lung lobes (right caudal, middle and cranial) were removed and fixed in 10% neutral buffered formalin. Left caudal lung lobe was aseptically removed for the measurement of bacillary load. A portion of left cranial lung lobe was stored in RNA *later®* (Ambion) at −20°C for isolation of RNA to be used for microarray and real time RT-PCR studies.

For histopathological examination, as described previously [31] sections of 5 μm thickness from formalin fixed and paraffin embedded tissues were cut on to glass slides and stained with haematoxylin and eosin. The percent granuloma in lung, type and extent of necrosis, organization of granuloma along with the type of infiltrating cells were assessed. In order to determine the extent of collagen deposition and fibrosis, the lung sections were also stained with Van Gieson stain.

### Bacterial enumeration

Specific portions of lungs were weighed and homogenized separately in 5 ml saline in a Polytron homogenizer. Appropriate dilutions of the homogenates were inoculated on to MB7H11 agar plates in duplicates and incubated at 37°C in a CO_2_ incubator for three to four weeks. The number of colonies were counted and expressed as log_10_ CFU/g of tissue. The detection limit in case of both lungs CFU was 1.0 log_10_ CFU/g.

### Labeling of RNA samples and quality control for 44 K microarray hybridization

To evaluate the gene expression profile associated with pulmonary tuberculosis, total RNA was isolated from lung tissues of *M. tuberculosis* infected and naive control guinea pigs (n = 3) by using Qiagen’s RNeasy mini kit as per the manufacturer’s recommendations followed by assessment of quality (specific activity) and quantity (yield) by using Bioanalyzer (Agilent Technologies). RNA samples then were labelled with Cy3 by using Agilent Quick-Amp labeling Kit as per the manufacturer’s recommendations. Briefly, 500 ng each of the control and test RNA samples were incubated with reverse transcription mix at 40°C and converted to double stranded cDNA primed by oligo dT with a T7 polymerase promoter. Synthesized double stranded cDNA was then used as template for cRNA generation. cRNA was generated by *in vitro* transcription along with the incorporation of Cy3 CTP during this step (Agilent Technologies). By using Qiagen’s RNeasy mini kit, fluorescently labeled cRNA was purified followed by the assessment of quality and quantity by using Bioanalyzer (Agilent Technologies).

### Hybridization and scanning

Linear amplified Cy3 labeled cRNA were hybridized to guinea pig 44 K microarray. Briefly, 1.65 μg of Cy3 labeled cRNAs were fragmented and hybridized to the array. Fragmentation of labeled cRNA and hybridization were carried out by using the Agilent Gene Expression Hybridization kit. Hybridization was carried out in Agilent’s Surehyb Chambers at 65°C for 16 hours. Following hybridization, the slides were washed by using Agilent Gene Expression wash buffers and scanned at 3μm resolution by using the Agilent Microarray Scanner G2505C under conditions to limit saturation to < 80% and were saved as TIFF images. The features then were extracted with the Feature Extraction Software (Agilent technologies, v10.7). Details of design, development and annotation of microarray are provided in Supporting materials and methods in Additional file 1.

### Microarray data analysis

The data was analyzed by using GeneSpring GX v11.5 software (Agilent Technologies). Normalization of the data was carried out in GeneSpring GX by using the 75^th^ percentile shift. It subtracts this value from the expression value of each entity and normalizes to specific control samples and fold change was calculated for the infected group relative to the baseline control (cRNA derived from uninfected animals). The fold intensity data then were filtered for significantly regulated (up and down regulated) genes in the treatment group in comparison to the control group based on the following stringent criteria: Expression fold values are provided in terms of log base 2. For up regulated genes, the cutoff used is fold change > 0.6 along with geometric mean fold change > 1 and Flags "detected" in infected samples. For filtering the down regulated genes, the cutoff used is fold change < − 0.6 along with geometric mean fold change < −1 and Flags "detected" in normal uninfected control sample that means a change in expression by ≥ 1.5 fold (up or down). Based on these criteria, only those genes, which showed a significant regulation in triplicates, were further subjected to hierarchical clustering based on Pearson coefficient correlation algorithm to identify significant gene expression patterns. Genes were classified on the basis of functional categories and pathways by using GeneSpringGX software and Genotypic Biointerpreter Biological Analysis software (Genotypic Technology Pvt. Ltd.). The microarray data reported here have been submitted at NCBI’s Gene Expression Omnibus [GEO Accession number: GSE32447].

### Real time RT-PCR

For the validation of 44 K GPOM, expression of a few selected genes was analysed by real time RT-PCR by employing SYBR green PCR Master Mix (Applied Biosystems) on the same RNA samples, which were used for the microarray study. Sequences of the primers employed for real time RT-PCR of C3AR1, CAMP, CCL5, IFNγ, C4BPA and 18S rRNA genes and comparison of gene expression pattern with respect to microarray are described in Supporting Table S2 in Additional file 1. For the analysis of real time PCR data ΔΔCt method was employed. First, ΔCt value was calculated for each sample as the difference between the Ct values for the gene of interest and the housekeeping gene (18S) in each sample. Then, ΔΔCt value was calculated as the difference between the ΔCt values of an experimental sample and the control sample. Fold change in gene expression were calculated as 2 ^-ΔΔCt^.

### Statistical analysis

Mean differences for percent fold induction in mRNA expression levels were analyzed by student’s t test. Differences were considered statistically significant, when *p* < 0.05. Based on Pearson coefficient correlation algorithm, the samples were clustered by using hierarchical clustering to identify similar conditions.

## Abbreviations

(GPOM): Guinea pig oligonucleotide microarray; (TB): Tuberculosis; (RT-PCR): Reverse Transcription – Polymerase Chain Reaction.

## Competing interest

The authors have no financial or non-financial competing interests.

## Author contributions

RJ, BD and AKT conceived, designed and performed the experiments, analysed the data and wrote the manuscript. All authors read and approved the final manuscript.

## Supplementary Material

Additional file 1 Supporting Materials and Methods and Tables. Click here for file

Additional file 2 ** Pulmonary gene expression signature of guinea pigs at 10 weeks post *M. tuberculosis* infection. ** The figure depicts the clustered heat maps for all the genes on the 44 K GPOM in case of infected guinea pigs compared to uninfected control. By using unsupervised hierarchical clustering algorithm, the most similar expression profiles are joined together to form a group. These are further joined in a tree structure, until all data forms a single group. Clustering is based on averaged distance between two clusters, which is the average of the pair-wise distance between entities in the two clusters. For measurement of similarity between conditions, Pearson coefficient correlation clustering algorithm is used. The color scheme for the hierarchical clustering is - yellow: no change in expression, magenta: higher expression in infected lungs relative to normal lungs and green: lower expression in infected samples relative to normal uninfected lungs. 1: Uninfected control; 2: Infected Lung 1; 3: Infected Lung 2; 4: Infected Lung 3. Click here for file

Additional file 3 ** List of genes exhibiting significant transcriptional regulation in the lungs of *M. tuberculosis *infected guinea pigs compared to uninfected guinea pigs.** The excel data sheets depict list of up and down regulated unique genes, for which, all the probes exhibited consist expression pattern. The expression values from multiple probes were averaged for a gene. Click here for file

Additional file 4 ** Pathway analysis for the up and down regulated genes.** The excel file describes the pathway analysis for the up and down regulated genes. The pathway analysis was performed by using Human Biointerpreter tool (http://genotypic.co.in/biointerpreter.html) based on KEGG database. Click here for file

Additional file 5 ** Comparison of pulmonary transcriptional response of guinea pig, human, non-human primate and mouse to *M. tuberculosis* infection.** The excel file depicts the comparison of up and down regulated genes from this guinea pig microarray study with those obtained from human, non-human primate and mouse microarray data available in the public database. The Venn diagrams depict the number of genes overlapping among different species. Click here for file
