# Supplementary material for: Development of the first oligonucleotide microarray for global gene expression profiling in guinea pigs: defining the transcription signature of infectious diseases
Source: BMC Genomics. 2012 Oct 2;13:520. doi: 10.1186/1471-2164-13-520 (PMC3475082; doi:10.1186/1471-2164-13-520)
Supplement: Additional file 1 — Supporting Materials and Methods and Tables. [file 1471-2164-13-520-S1.doc]

**Additional Data File 1**

**Supporting Materials and Methods**

**Design and production of microarray**

Design and production of guinea pig microarray, microarray processing for various samples and data analysis was carried out at Genotypic Technology Pvt. Ltd, Bangalore. Development of 44K array is based on a 244K microarray, which was initially designed to contain ~60-mer oligonucleotide probes from multiple species. The 244K array comprised of a total number of 2,43,504 features including 2,13,039 probes from different mammalian species, 2105 Agilent controls and 28,360 blank spots. The number of features from various mammals that have been used for designing the 244K multi-species array is detailed in **Supporting Table S1**. All the oligonucleotides were designed and synthesized *in situ* as per the standard algorithms and methodologies used by Agilent Technologies for 60- mer *in situ* oligonucleotide DNA microarray.

**RNA and Genomic DNA isolation**

RNA and genomic DNA used for the design and development of guinea pig microarray were isolated from various tissues such as lung, liver, spleen, brain, muscle, kidney and bone marrow of 6-8 weeks old guinea pigs. Genomic DNA was extracted from guinea pig tissues by using DNeasy Blood and Tissue Kit as per the manufacturer’s instructions (Qiagen). Total RNA was isolated from various tissues by using Agilent Total RNA Isolation Mini kit as per the manufacturer’s instructions (Agilent Technologies)

**DNA and RNA Quality Control**

Quality and quantity of genomic DNA were determined by using agarose gel electrophoresis and Agilent Nanodrop spectrophotometer (Agilent Technologies). Intact genomic DNA with A_260/280_ ≥ 1.8 and A_260/230_ ≥ 1.0 was used for microarray hybridization experiments. RNA concentration and purity was also determined by using the Nanodrop® ND-1000 spectrophotometer (NanoDrop Technologies) and the integrity of RNA was verified on an Agilent 2100 Bioanalyzer by using the RNA 6000 Nano LabChip (Agilent Technologies). RNA samples with an rRNA 28S/18S ratio ≥ 1.5, rRNA contribution ≥ 30%, RNA integrity number (RIN) ≥ 6, A_260/280_ > 1.8 and A_260/230_ ≥ 1.3 were used for the microarray experiments.

**RNA and DNA labeling and microarray hybridization**

Total RNA isolated from various guinea pig tissues (lung, liver, spleen, brain, muscle, kidney and bone marrow) was amplified by using the Agilent Low RNA Input Fluorescent Linear Amplification Kit and labelled with Cy3 CTP using Agilent Quick Amp Kit PLUS. RNA was then reverse transcribed to double stranded cDNA by using oligo dT primers. The double stranded cDNA was then used as template for cRNA generation by *in vitro* transcription. Labeled cRNA was purified and its quality was assessed for yield and specific activity by using Agilent Nanodrop spectrophotometer (Agilent Technologies).

Genomic DNA was labeled with Cy5 dUTP by using Agilent Genomic DNA Labeling Kit PLUS. Briefly, 1.5μg of genomic DNA was digested at 37°C with restriction enzymes Alu I and Rsa I and labeled with dUTP dyes. The labeled RNA and DNA were then purified and assessed for yields and specific activity. Cy3 labelled cRNA and Cy5 labelled genomic DNA showed a specific activity of 17.57 pmol dye/μg cRNA and 30.35 pmol dye/μg DNA, respectively.

The 244K microarray was then hybridized with Cy3 labelled cRNA produced from pooled RNA obtained from various guinea pig tissues and Cy5 labelled genomic DNA by using standard Agilent *in situ* Hybridization kit protocol. Hybridization was carried out in Agilent’s Surehyb Chambers at 65º C for 16 hours. The hybridized slides were washed by using Agilent Gene Expression wash buffers and scanned by using the Agilent Microarray Scanner G Model G2565BA at 5μm resolution. Similarly, 5 μg of Cy5 labeled DNA was fragmented by using Comparative Genomic Hybridization kit of Agilent and hybridized on the same array for 40 hours at 65º C, washed and scanned as described earlier. Data was extracted from the images by using Feature Extraction software (v9.5) from Agilent.

Based on the intensities observed in the above hybridization experiment, the probes showing signal intensity twice or more than twice the background signal intensity were selected and a 44K array was designed. As described above, all the oligonucleotides were designed and synthesized *in situ* as per the standard algorithms and methodologies used by Agilent Technologies.

# Annotation of probes for 44K microarray

The guinea pig 44K microarray comprised of a total number of 45220 features including 29846 valid features from different mammalian species, 1132 probes for guinea pig transcripts and 12825 probes for guinea pig ESTs, 1264 Agilent positive controls and 153 Agilent negative controls. The % representation of a particular species is calculated with respect to the total number of probes in the array. Upon sequence analysis, 42,511 probes were found to be unique. Of the 42,511 unique probes, 20,574 represented Reference Sequences (REFSEQ Database), 86 probes corresponded to Agilent proprietary transcript database, 18,914 probes represented EST database and 2,284 probes represented transcripts unique to ENSEMBLE and TIGR database. All the guinea pig specific probes were re-annotated by blast based homology to the transcript sequence database of guinea pig from NCBI (as available in Sep 2011), with a criterion of best hit of more than 75% identity over a minimum 30 base pair alignment length. 2971 of the guinea pig specific probes were found to correspond to 344 unique genes of guinea pig.

Further, for biological interpretation, homolog or gene ontology annotation was also obtained for all the probes by blast based homology to reference sequence database of Human, Mouse and Rat. The homolog annotation for the probes is based on the criteria of best hit of more than 75% identity over a minimum 30 base pair alignment length in any of the three organisms, human, mouse or Rat. Annotated genes that exhibited significant up or down regulation (4190 genes) were included for functional analysis and are listed in **Additional Data File 3.** Functional classification of differentially regulated genes was carried out by using GeneSpringGX software and Genotypic Biointerpreter Biological Analysis software (Genotypic Technology Pvt. Ltd.). The microarray data reported here have been submitted at NCBI’s Gene Expression Omnibus [GEO Accession number: GSE32447].

**Supporting Tables**

| **Supporting Table S1. Probe distribution in 244K GPOM:** The table depicts the number of features derived from various mammalian species that have been used for designing the 244K GPOM. | | |
| --- | --- | --- |
| **Organism** | **Sequence data source** | **Number of Probes in 244K Array** |
| Human (*Homo Sapiens*) | Agilent Catalogue Arrays | 36,866 |
| Mouse (*Mus musculus*) | *Agilent Catalogue Arrays* | 40,896 |
| Rat (*Rattus norvegicus)* | Agilent Catalogue Arrays | 39,381 |
| *Rhesus Monkey (Macaca mulatta)* | Agilent Catalogue Arrays | 20,217 |
| *Dog (Canis familiaris)* | Agilent Catalogue Arrays | 42,034 |
| Horse (*Equus caballus*) | NCBI, mRNA sequences | 1120 |
| Cat (*Felis catus*) | NCBI, mRNA sequences | 637 |
| Sheep (*Ovis aries*) | NCBI, mRNA sequences | 1356 |
| Pig (*Sus scrofa*) | NCBI, mRNA sequences | 16,003 |
| Guinea pig (*Cavea porcellus*) | NCBI, mRNA sequences | 1132 |
| *Chinchilla lanigera* | NCBI, mRNA sequences | 84 |
| Chimpanzee (*Pan troglodyte*) | NCBI, mRNA sequences | 849 |
| Gray tailed opussum (*Monodelphis domestica*) | NCBI, mRNA sequences | 224 |
| Cattle (*Bos Taurus*) | NCBI, mRNA sequences | 12,240 |
|  | **Total Number of probes** | **2,13,039** |
|  | | |

| **Supporting Table S2. Quantitative real time RT-PCR for microarray validation.** The table depicts the primer sequences employed for real time RT-PCR of C3AR1, C4BPA, CAMP, CCL5, IFN-γ and 18S rRNA genes and the gene expression pattern observed with respect to that observed in case of microarray. | | | | | | | |
| --- | --- | --- | --- | --- | --- | --- | --- |
| Primer Name |  | **Primer Sequence** | **Primer Length (bases)** | Tm (º C) | **Amplicon size (base pairs)** | **Expression profile** | **Match with Microarray** |
| **C3AR1** | F | TCTGTATCTCCCTTATTTCACT | 22 | 60 | 143 | Down | Match |
|  | R | CCTAAGTGCCCATCAACAGA | 20 | 60 |  |  |  |
| **C4BPA** | F | GCCTGGATGCTTTGCTGTC | 19 | 60 | 162 | Up | Match |
|  | R | CAAATATACGAGGCACAGATG | 21 | 60 |  |  |  |
| **CAMP** | F | CGCGAGTACGGGCAGATC | 18 | 60 | 163 | Down | Match |
|  | R | GGCAAGAACACACTAGGTAG | 20 | 60 |  |  |  |
| **CCL5** | F | CTGGCCCACTGCTTAGCAAT | 20 | 60 | 62 | Down | Match |
|  | R | CCTTGCTTCTTTGCCTTGAAA | 21 | 60 |  |  |  |
| **IFNγ 0 18S CT0** | F | ACAAGGTGCAGGCTTTCAAAA | 21 | 60 | 65 | Down* | Match |
|  | R | TTGGCGCTGGACATGCT | 17 | 54 |  |  |  |
| **18S CT0** | F | TGCATGGCCGTTCTTAGTTG | 20 | 60 | 76 | For normalization | |
|  | R | AGTTAGCATGCCAGAGTCTCGTT | 23 | 68 |  |  |  |
| * While one of the samples showed mild down regulation other showed no change by real time RTPCR. By microarray overall expression for IFNγ showed mild down regulation. | | | | | | | |

**References:**
