# Supplementary figures and images for: Development of the first oligonucleotide microarray for global gene expression profiling in guinea pigs: defining the transcription signature of infectious diseases
Source: BMC Genomics. 2012 Oct 2;13:520. doi: 10.1186/1471-2164-13-520 (PMC3475082; doi:10.1186/1471-2164-13-520)

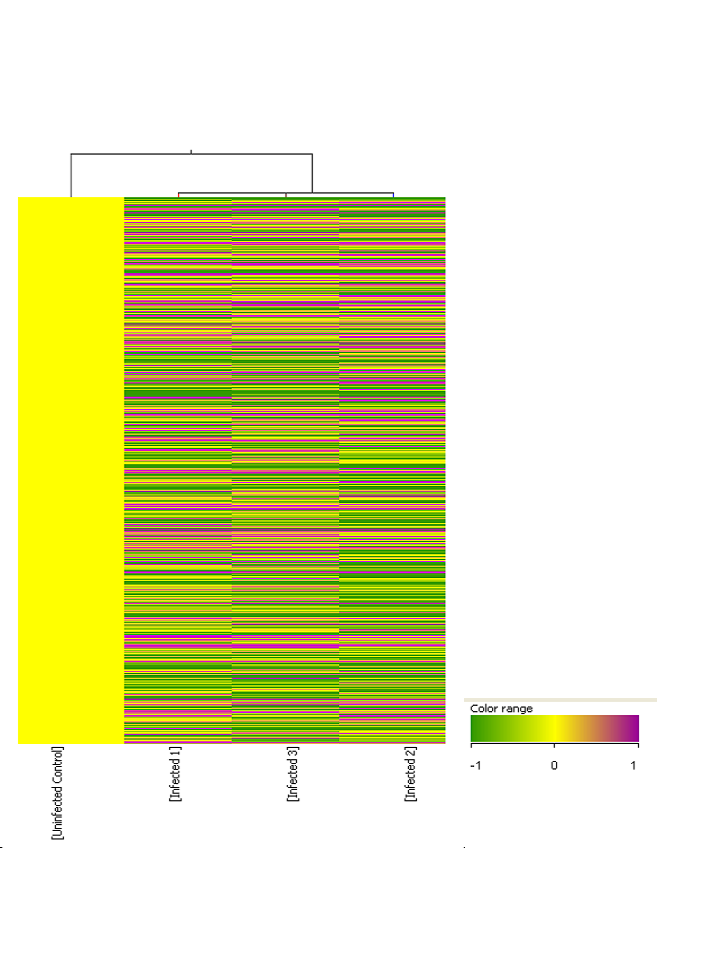

Supplement: Additional file 2 — Pulmonary gene expression signature of guinea pigs at 10 weeks post M. tuberculosis infection. The figure depicts the clustered heat maps for all the genes on the 44 K GPOM in case of infected guinea pigs compared to uninfected control. By using unsupervised hierarchical clustering algorithm, the most similar expression profiles are joined together to form a group. These are further joined in a tree structure, until all data forms a single group. Clustering is based on averaged distance between two clusters, which is the average of the pair-wise distance between entities in the two clusters. For measurement of similarity between conditions, Pearson coefficient correlation clustering algorithm is used. The color scheme for the hierarchical clustering is - yellow: no change in expression, magenta: higher expression in infected lungs relative to normal lungs and green: lower expression in infected samples relative to normal uninfected lungs. 1: Uninfected control; 2: Infected Lung 1; 3: Infected Lung 2; 4: Infected Lung 3. [file 1471-2164-13-520-S2.tiff]
